# Retrospective evaluation of clinical use of cis-atracurium in horses

**DOI:** 10.1371/journal.pone.0221196

**Published:** 2019-08-15

**Authors:** Alexandru Tutunaru, Julien Dupont, Alexandra Gougnard, Keila Ida, Didier Serteyn, Charlotte Sandersen

**Affiliations:** Department of Clinical Sciences, Faculty of Veterinary Medicine, University of Liège, Liège, Belgium; Public Library of Science, UNITED KINGDOM

## Abstract

**Background:**

To the authors’ knowledge, there are no reports describing the use of cis-atracurium in the horse.

**Objective:**

To describe the onset time and the duration of the neuromuscular blockade (NMB) of three different doses of cis-atracurium in horses and to determine the appropriate dose needed maintain a NMB.

**Study design:**

Retrospective study.

**Methods:**

Horses which received cis-atracurium as part of a balanced anaesthetic protocol at the Equine Teaching Hospital of the University of Liège between March 2014 and June 2017 were included in this study. A train-of-four (TOF) stimulation pattern was used to assess the NMB. The cis-atracurium induction dose, the onset and duration of action (when TOF count was under three twitches) of the first bolus, the number of supplementary boluses of cis-atracurium and the total dose of cis-atracurium administered per horse, the total duration of the NMB and the recovery time were recorded and analysed. Also the use of an antidote and any side effects produced by cis-atracurium were recorded.

**Results:**

From 37 horses that received cis-atracurium during this period, only 23 had a complete records and were included in the study. Three different doses of cis-atracurium were used to induce NMB: 100 μg/kg (*n* = 8) 75 μg/kg (*n* = 3) and 50 μg/kg (*n* = 12). Cis-atracurium 50 μg/kg failed to induce NMB in 3 horses. The onset of action was not significantly different between the three doses (5 minutes). The duration of the NMB was dose-dependent. The calculated dose of cis-atracurium necessary to maintain a NMB was 2.3 μg/kg/minute based on the sum of the induction dose and the supplementary boluses divided by the duration of the NMB.

**Main limitations:**

A further prospective study is needed to confirm the results.

**Conclusions:**

Cis-atracurium can be an alternative to other NMBA in horses.

## Introduction

Neuromuscular blocking agents (NMBAs) provide profound muscle relaxation and are used as part of a balanced anaesthetic protocol. Along with hypnosis and analgesia, muscle relaxation is one of the elements of the general anaesthesia triad. Atracurium is a non-depolarising, benzylisoquinolinium family compound that may trigger histamine release when injected rapidly or in high doses. Cis-atracurium is one of the 10 isomers of atracurium and it is five times more potent compared to atracurium. As a result, cis-atracurium is less likely to produce histamine release at equipotent dosage [1].

Many other NMBAs have already been studied in horses [1] and the use of cis-atracurium is documented in dogs and pigs [2,3]. To the authors’ knowledge, there is no study on the clinical use of cis-atracurium published in this species. However, a review article mentions the use of cis-atracurium in horses and suggested a dose rate that was tested in an unpublished study [1]. The aim of this retrospective study was to assess the neuromuscular blockade following single or multiple boluses of cis-atracurium in horses and to evaluate any adverse reactions produced by this NMBA.

## Materials and methods

This is a retrospective study consisting of analysis of anaesthetic records. The owners signed an agreement for the use of the scientific data obtained from their horses.

### Inclusion and exclusion criteria

A retrospective analysis was performed to review the anaesthetic record database of the Equine Teaching Hospital of the University of Liège for horses that had received intravenous (IV) cis-atracurium (Nimbex, Glaxo Smith Kline Pharmaceuticals, Wavre, Belgium) as part of a balanced anaesthetic protocol between March 2014 and June 2017. Only horses with comprehensive anaesthetic records were included in this study. Horses were excluded from the study if the anaesthetic record was incomplete, if the neuromuscular block was not monitored using a peripheral nerve stimulator or if there were no data regarding the first 24 hours of the postoperative period.

### Monitoring during anaesthesia

A standard anaesthetic protocol was adapted individually to each horse. Cardiovascular (ECG, arterial blood pressure and pulse oximetry) and respiratory (capnography, oxygen and alveolar anaesthetic agent concentration) parameters were monitored continuously and recorded every five minutes. Arterial blood samples were analysed and pH, PaCO_2_ (arterial partial pressure of CO_2_), PaO_2_ (arterial partial pressure of O_2_) and electrolyte plasma level (Cobas B 123, Roche Diagnostics International Ltd, Rotkreuz, Switzerland) were recorded every 30 minutes.

### Neuromuscular blockade

Two needle electrodes were placed over the left or right superficial peroneal nerve, according to the type of surgery, and train-of-four (TOF) stimulation pattern was used to assess the neuromuscular blockade (NMB). The use of a calibrated acceleromyography (TOF-Watch S, Organon Ltd, Dublin, Ireland) enabled an objective evaluation of the TOF ratio (the ratio between the last and the first twitch) and TOF count. The acceleromyograph crystal was taped over the tip of the hoof. The TOF results were recorded every 15 seconds on a specific NMB record.

The data regarding the use of cis-atracurium consisted of: cis-atracurium induction dose (μg/kg), the onset of action (from cis-atracurium injection to twitch count < 3, in minutes), any supplementary cis-atracurium doses administered (μg/kg), the NMB duration after the first bolus (the total time when TOF count was < 3 twitches, in minutes), the total dose of cis-atracurium administered during anaesthesia (μg/kg) and the total duration of the NMB (when TOF count is under 3 twitches, in minutes), the use of a NMB reversal agent, the recovery time (from cis-atracurium injection to TOF rate over 90%, in minutes) and any side effects of cis-atracurium (histamine release, bronchospasm or laudanosine induced epileptic seizures) during anaesthesia or in the first 24 hours of the postoperative period). The cis-atracurium induction dose was determined by each anaesthetist depending on personal preference and the duration of surgery. The total average dose of cis-atracurium administered per horse (μg/kg) was divided by the total duration of the NMB (minutes) to obtain the dose of cis-atracurium in μg/kg/minute.

### Data analysis

Analyses of variance were used to compare the onset and duration of action of cis-atracurium (adjusted for onset) between the three dose rates (PROC GLM; SAS Version 9.3, SAS Institute, Inc., NC, USA). Normality assumption of the distribution of the error terms was confirmed by the use of four tests (PROC UNIVARIATE). Differences were considered significant if the *p* value <0.05. T-test was used to compare heart rate and mean arterial blood pressure 5 minute before and 5 minutes after Cis-atracurium injection. Data are presented as mean ± standard deviation or median (range).

## Results

### Selected population

Cis-atracurium was administered in 37 horses. Only 23 records were complete and were included in this retrospective study. All collected data is provided in S1 Table. The age and weight were 147 ±75 months (mean ± SD) and 497 ±127 kg (mean ± SD), respectively, and there were 12 mares, nine geldings and two stallions. Twenty-two horses were classified ASA 2 and one as ASA 3 based on the ASA (American Society of Anesthesiologists) physical status classification system. Twenty-one horses were anaesthetized for ophthalmic surgical procedures and two for orthopaedic procedures.

### Anaesthetic protocol

Intravenous (IV) flunixin meglumine (Emdofluxin 50, Emdoka bvba, Hoogstraten, Belgium) 1.1 mg/kg was administered to all horses before anaesthetic induction. Acepromazine (Placivet, KELA N.V., Hoogstraten, Belgium) (0.094 ±0.018 mg/kg) was administered to 21 horses either intramuscularly (IM) (*n* = 17) or IV (*n* = 4) 30 minutes before the anaesthetic induction. Five minutes before induction, either xylazine (Proxylaz, Prodivet pharmaceuticals, Eynatten, Belgium) 0.6 mg/kg IV (*n* = 22) or romifidine (Sedivet, SCS Boehringer Ingelheim Comm.V,Bruxelles, Belgium) 0.04 mg/kg IV (*n* = 1) were administered. Midazolam (Dormicum, Roche Diagnostics International Ltd, Rotkreuz, Switzerland) 0.06 mg/kg and ketamine (Ketamidor, Richter Pharma, Wels, Austria) 2.2 mg/kg were injected intravenously to induce anaesthesia in all horses. After tracheal intubation the endotracheal tube was connected to a rebreathing circuit and volume controlled ventilation was initiated using the Tafoniusanaesthetic machine (Vetronic servicies Ltd, Abbotskerswel, UK). Isoflurane (IsoFlo, Zoetis, Louvain-la-Neuve, Belgium) in oxygen-enriched air was used to maintain general anaesthesia. The following drugs were administered during anaesthesia to provide analgesia or to increase depth of anaesthesia: ketamine (*n* = 13), morphine (Morphine HCl Sterop, Laboratoires Sterop, Bruxelles, Belgium) *(n* = 9), xylazine either by a continuous rate infusion (CRI) or as a bolus (*n* = 6), romifidine CRI (*n* = 1), butorphanol (Butomidor, Richter Pharma, Wels, Austria) (*n* = 1). A dobutamine (Dobutrexmylan, Mylan, Hoeilaart, Belgium) CRI (*n* = 18) was administered to maintain a mean arterial blood pressure over 70 mmHg. No other inotropes or vasopressors were administered. Lactate Ringer solution (Ringer’s Lactate, Baxter, Bruxelles, Belgium or Vetivex, Dechra limited, Antwerp, Belgium) was infused throughout anaesthesia.

The duration of general anaesthesia (from induction to the end of isoflurane administration) and surgery was 137 ± 43 (mean ± SD) minutes and 82 ± 29 minutes (mean ± SD), respectively.

An association of sodium penicillin (Penicilline, KELA N.V., Hoogstraten, Belgium) (22000 UI/kg IV) with gentamicin (6.6 mg/kg IV) (*n* = 11) and procaine penicillin (Peni-Kel, KELA N.V., Hoogstraten, Belgium) (22000 UI/kg IM) with gentamicin (Genta-Equine, Franklin Pharmaceuticals Limited, Meath, Irland) (6.6 mg/kg IV) (*n = 10*) was administered. One horse received only sodium penicillin (22000 UI/kg IV) and a second horse received only Cefquinome (Cobactan, Emdoka bvba, Hoogstraten, Belgium) (1 mg/kg IV).

### Neuromuscular blockade

Three doses of cis-atracurium were administered: 100 μg/kg, 75 μg/kg and 50 μg/kg. Cis-atracurium 100 μg/kg IV was administered to 8/23 horses (35%) and the median onset of action was 3.5 (2–6) minutes. TOF count was maintained under three twitches for 32.5 minutes (28–44) after this bolus. The mean recovery time in 5 of 8 horses was 57 minutes (44–69). The recovery time of the other 3 horses was not taken in consideration since they received additional boluses of cis-atracurium to prolong the duration of the NMB. Cis-atracurium 75 μg/kg IV was administered to 3/23 horses (13%) and the median onset of action was 5 minutes (2–10). TOF count was maintained under three twitches for 25 minutes (22–40) after the bolus. The recovery time was 60 minutes (60–73). Cis-atracurium 50 μg/kg IV was administered to 12/23 horse (52%) and the median onset of action was 5 minutes (3–8). TOF count was maintained under three twitches for 12.5 minutes (0–23) after the bolus. However, cis-atracurium at 50 μg/kg failed to decrease the TOF count under three in 3/12 (25%) horses and additional doses were needed. Only two out of 12 horses did not receive supplementary boluses to increase the duration of the NMB and the recovery time was 39 and 44 minutes, respectively (Table 1).

**Table 1:** The onset, duration of action and recovery time after intravenous administration of cis-atracurium at three different dose rates. Results are expressed as median (range).

Cis-atracurium dose (μg/kg)	No of horses	Onset of action (minutes)	NMB duration after the first bolus of cis-atracurium (minutes)	**Recovery time (minutes)
100	8	3.5 (2–6)	32.5 (28–44)	57 (44–69) (n = 5)
75	3	5 (2–10)	25 (22–40)	60 (60–73) (n = 3)
50	12	5 (3–8) *	12.5 (0–23)	39 and 44 (n = 2)

* Cis-atracurium failed to induce neuromuscular blockade (twitch count under 3) in 3 horses.

** Includes only horses that did not received supplementary boluses of cis-atracurium

The onset of action was not significantly different between the three doses of cis-atracurium (p<0.05). The duration of action of cis-atracurium at a dose of 100μg/kg was not significantly different from 75μg/kg (p<0.05). However, it was significantly shorter when 50 μg/kg dose was administered compared to the other two dose rates (p<0.05).

Based on the average dose of cis-atracurium administered (100 μg/kg) and the average duration of the NMB (44 minutes) the calculated dose of cis-atracurium needed to maintain the TOF count under three twitches was 2.3 μg/kg/minute.

Neostigmine (Prostigmine, Meda Pharma, Bruxelles, Belgium) 15 μg/kg IV was administered in 15/23 horse (65%) from which 13 (87%) also received atropine (Atropine Sulfate Sterop)^k^ 15 μg/kg IV. Neostigmine was administered when TOF ratio was over 90% and so neostigmine administration did not influence the recovery time compared to those who did not receive neostigmine. The decision to administer neostigmine with or without atropine was made by the anaesthetist and based on his personal preference.

Side effects suspected to be produced by cis-atracurium (histamine release, bronchospasm or laudanosine induced epileptic seizures) were not observed at any dose. Heart rate and arterial blood pressure did not appear to change after the induction of the NMB regardless the dose used. However, these data were not analysed statistically throughout the duration of the procedure.

## Discussion

The aim of this study was to determine which dose of cis-atracurium produces moderate and predictable NMB (TOF count under three twitches, described as ‘surgical relaxation’ in horses) [1]. Fuchs-Buder (2007) defines the moderate NMB in humans ‘as the time period from the reappearance of the first twitch (T1) until the reappearance of the fourth twitch’ (T4) [4]. This retrospective study also evaluated the differences between three doses of cis-atracurium in relation to the onset and duration of action and the time needed to recover. The onset of action was not significantly different between the three doses (p>0.05). However, in three of 12 horses, cis-atracurium at 50 μg/kg failed to induce NMB. As suspected, the duration of action was directly associated to the dose of cis-atracurium administered. Cis- atracurium 100 and 75 μg/kg produced a longer duration of action compared to cis-atracurium 50 μg/kg (p<0.05).

### Other NMBAs that might be used in horses

Depolarising NMBAs, such as succinylcholine, are used for rapid sequence intubation on anaesthetised human patients to facilitate intubation [5]. Horses are easily intubated compared to humans and such drugs are of little value in this species. In addition, succinylcholine may produce severe side effects such as cardiac dysrhythmias, muscle fasciculation and hyperkalemia [6]. Nondepolarising NMBAs are free of such side effects. Aminosteroids (eg. rocuronium, vecuronium) and benzoquinolines (eg. atracurium, cis-atracurium) are the two main classes of nondepolarising NMBAs. Rocuronium was successfully tested in horses for ophthalmic interventions [7]. Vecuronium was assessed in this species but the dose needed to produce predictable NMB was not determined [8]. Compared to benzoquinolines, aminosteroids, especially rocuronium, have the advantage of being fully reversible by the specific agent, sugammadex. However, the price of this reversal agent is prohibitive, especially in large species.

### Atracurium and cis-atracurium

Atracurium is a benzoquinoline compound with a rapid biotransformation, independent to the renal and hepatic function. It is metabolised by tissue esterase and by Hoffman elimination [1]. The use of atracurium was first described in healthy horses by Hildebrand and Arpin (1988) [9]. In ocular interventions, atracurium was compared to a control group in which F_I_Iso was increased to produce a central eyeball position. Atracurium produced less cardiovascular changes and a shorter recovery time compared to the control group [10]. The main side effect of atracurium is the ability to trigger histamine release, which can lead to hypotension [1]. In addition, one of the metabolites of atracurium, laudanosine, may induce seizures if administered in high doses. Side effects of atracurium have not been reported in horses yet [1]. Cis-atracurium is one of the 10 isomers of atracurium, with a more potent activity and fewer side effects in human patients [11]. Mean arterial blood pressure and heart rate were both compared 5 minutes before and after cis-atracurium injection. There were no statistical differences between this two time points (p = 0.179632 for mean arterial pressure; p = 0.488885 for heart rate) suggesting that no histamine release was produced. To the authors’ knowledge, the use of cis-atracurium has not been described in horses yet.

### Study limitations

Different antibiotics, such as aminoglycosides, have notable effects on NMB. The data from two of the horses not treated with gentamicin might influence the results. However, Hildebrand (1994) found minimal differences between the duration of action of atracurium in horses treated or not with gentamicin [12].

Body temperature may influence both the degradation of cis-atracurium and the response to the nerve stimulation [13]. Unfortunately, the influence of body temperature could not be analysed in the present study. Due to the retrospective nature of this study, we consider that the recorded temperature data were unreliable. Body temperature was recorded as rectal temperature by multiple assessors and with different thermometers. TOF Watch and TOF Watch S machines determine the TOF value with an algorithm that calculates the TOF ratio from the ratio of T4/T2 if the value of T2 is higher than the value of T1. If this ratio is also above 1.0, the TOF Watch S will only display 100%. In contrast, the TOF Watch SX machine does not have this special algorithm built-in and is more recommended in the research setting [4].

The number of horses which received cis-atracurium 75 μg/kg was small (3/23) and results must be regarded with caution. However, this is a retrospective evaluation of the use of cis-atracurium in horses and we considere that all data collected should be presented.

## Conclusion

The NMBAs are useful drugs in the context of balanced anaesthesia protocols. They are especially indicated for ophthalmological surgeries and to prevent involuntary movements during anaesthesia in the horses. Cis-atracurium seems to induce few side effects and may be an alternative to rocuronium to produce NMB in horses. Our results suggest the use of cis-atracurium 100 μg/kg IV and 2.3 μg/kg/minute for a successful induction and maintenance of a NMB, respectively. Due to some variations in the observed results, monitoring of NMB is recommended. A prospective study is further requested to confirm these results.

## Supporting information

S1 Table Complete data collected from the 23 records. (XLSX)
